# Branched-Chain Amino Acids Can Predict Mortality in ICU Sepsis Patients

**DOI:** 10.3390/nu13093106

**Published:** 2021-09-03

**Authors:** Alexander Christian Reisinger, Florian Posch, Gerald Hackl, Gunther Marsche, Harald Sourij, Benjamin Bourgeois, Kathrin Eller, Tobias Madl, Philipp Eller

**Affiliations:** 1 Intensive Care Unit, Department of Internal Medicine, Medical University of Graz, 8036 Graz, Austria; alexander.reisinger@medunigraz.at (A.C.R.); gerald.hackl@medunigraz.at (G.H.); 2 Department of Internal Medicine, Division of Oncology, Medical University of Graz, 8036 Graz, Austria; florian.posch@medunigraz.at; 3 Otto Loewi Research Center for Vascular Biology, Immunology and Inflammation, Division of Pharmacology, Medical University of Graz, 8036 Graz, Austria; gunther.marsche@medunigraz.at; 4 Department of Internal Medicine, Division of Endocrinology and Diabetology, Medical University of Graz, 8036 Graz, Austria; ha.sourij@medunigraz.at; 5 Gottfried Schatz Research Center for Cell Signaling, Metabolism and Aging, Molecular Biology and Biochemistry, Medical University of Graz, 8036 Graz, Austria; benjamin.bourgeois@medunigraz.at; 6 Department of Internal Medicine, Division of Nephrology, Medical University of Graz, 8036 Graz, Austria; kathrin.eller@medunigraz.at; 7 BioTechMed Graz, 8036 Graz, Austria

**Keywords:** sepsis, ICU, metabolomics, NMR spectroscopy, branched-chain amino acids, lipoproteins

## Abstract

Sepsis biomarkers and potential therapeutic targets are urgently needed. With proton nuclear magnetic resonance (^1^H NMR) spectroscopy, several metabolites can be assessed simultaneously. Fifty-three adult medical ICU sepsis patients and 25 ICU controls without sepsis were prospectively enrolled. ^1^H NMR differences between groups and associations with 28-day and ICU mortality were investigated. In multivariate metabolomic analyses, we found separate clustering of ICU controls and sepsis patients, as well as septic shock survivors and non-survivors. Lipoproteins were significantly different between sepsis and control patients. Levels of the branched-chain amino acids (BCAA) valine (median 43.3 [29.0–53.7] vs. 64.3 [47.7–72.3] normalized signal intensity units; *p* = 0.005), leucine (57.0 [38.4–71.0] vs. 73.0 [54.3–86.3]; *p* = 0.034) and isoleucine (15.2 [10.9–21.6] vs. 17.9 [16.1–24.4]; *p* = 0.048) were lower in patients with septic shock compared to those without. Similarly, BCAA were lower in ICU non-survivors compared to survivors, and BCAA were good discriminators for ICU and 28-day mortality. In uni- and multivariable logistic regression analyses, higher BCAA levels were associated with decreased ICU- and 28-day mortality. In conclusion, metabolomics using ^1^H NMR spectroscopy showed encouraging potential for personalized medicine in sepsis. BCAA was significantly lower in sepsis non-survivors and may be used as early biomarkers for outcome prediction.

## 1. Introduction

Sepsis is a common global life-threatening medical condition, and more than 31 million patients each year suffer from sepsis or septic shock [1]. The absolute number of sepsis patients is continuously increasing over the last years because of a more widespread use of immunosuppressive therapies, increasing age and the emerge of multi-resistant bacteria [2]. Sepsis is not simply the presence of bacteremia, but also triggered by the host’s response to the infection causing organ dysfunction. This concept is reflected in the current sepsis-3 definition [3]. The diagnosis of sepsis includes a suspected infection and the presence of a new organ dysfunction measured by the sequential organ failure assessment (SOFA) score. Septic shock represents a sicker subpopulation with an increased blood lactate level above 2 mmol/L and the need for vasopressor therapy to maintain a mean arterial pressure (MAP) over 65 mmHg. Apart from lactate, other biomarkers may be helpful in the early recognition and diagnosis of sepsis, in the assessment of disease severity, and for outcome prediction. Some inflammatory markers such as C-reactive protein (CRP) and procalcitonin (PCT) were not consistently able to predict the outcome of sepsis patients, and therefore better biomarkers are needed [4]. We were recently able to show that lipoprotein functionality parameters such as the arylesterase activity of high-density lipoprotein (HDL) associated paraoxonase 1 may be useful for outcome prediction in sepsis [5]. Therefore, we aimed to validate and extend these findings using proton nuclear magnetic resonance (^1^H NMR) spectroscopy. This emerging technique within the field of medicine, enables detection, identification, and quantification of metabolites and lipoproteins in serum or plasma samples with exceptionally good reproducibility and robustness [6,7]. ^1^H NMR can be used to assess samples for several metabolites simultaneously, providing the so-called metabolomic profile or metabolome. The advantage of this evolving method is the option of either a targeted or an untargeted approach (“global metabolomics”), making the identification of metabolites that were unknown to play a role in the investigated disease possible [8]. Furthermore, changes within the genome, transcriptome and the proteome can be displayed within the metabolome, thus providing a direct read-out for the functional alterations associated therewith.

In this study, we applied metabolomic methods using ^1^H NMR spectroscopy to analyze metabolites and lipoprotein-derived parameters from patients with sepsis and septic shock admitted to the intensive care unit (ICU) as well as ICU patients (controls) without sepsis or bacteremia to identify biomarkers associated with ICU outcome.

## 2. Materials and Methods

### 2.1. Study Population and Study Design

We recruited patients older than 18 years with sepsis and septic shock and ICU controls admitted to the ICU of the Department of Internal Medicine at the Medical University of Graz, Austria, as formerly published [5]. In brief, sepsis-3-criteria were applied to define sepsis patients [3], and the control group consisted of consecutive ICU patients without sepsis or bacteremia at the time of sampling. The study protocol was approved by the Institutional Review Board (IRB) of the Medical University of Graz, Austria (30–258 ex 17/18) and complied with the Declaration of Helsinki. Written informed consent was obtained from all conscious participants. In comatose non-survivors, the IRB waived the need for written informed consent.

### 2.2. Laboratory Analyses

Blood cell count, serum creatinine, bilirubin, CRP, PCT, and interleukin-6 (IL-6) were measured using a Sysmex (Sysmex Austria GmbH, Vienna, Austria) or Cobas (Roche Diagnostics, Mannheim, Germany) analyzer as appropriate. For further analyses, we centrifuged peripheral blood samples at 3000 rpm for 10 min and stored aliquots at −80 °C without any thawing/freezing cycles. Metabolomic analyses were performed in batch to avoid any inter-assay variance.

### 2.3. Reagents

Sodium phosphate, dibasic (Na_2_HPO_4_), sodium hydroxide (NaOH), hydrochloric acid (HCl, 32% m/v), and sodium azide (NaN_3_) were obtained from VWR International (Darmstadt, Germany). 3(Trimethylsilyl) propionic acid-2,2,3,3-d_4_ sodium salt (TSP) was obtained from Alfa Aesar (Karlsruhe, Germany). Deuterium oxide (D_2_O) was obtained from Cambridge Isotopes Laboratories (Tewksbury, MA, USA). Deionized water was purified using an in-house Milli-Q Advantage Water Purification System from Millipore (Schwalbach, Germany). All chemicals were used without further purification. The phosphate NMR buffer solution was prepared by dissolving 5.56 g of anhydrous Na_2_HPO_4_, 0.4 g of TSP, and 0.2 g NaN_3_, in 400 mL of D_2_O and adjusted to pH 7.4 with 1 M NaOH and HCl. Upon addition of D_2_O to a final volume of 500 mL the pH was re-adjusted to pH 7.4 with 1 M NaOH and HCl.

### 2.4. Lipoprotein Quantification Using NMR

Blood serum lipoproteins were analyzed on a Bruker 600 MHz Avance Neo NMR spectrometer using the Bruker IVDr lipoprotein subclass analysis protocol. Serum samples were thawed, and 330 µL of each sample mixed with 330 µL of Bruker serum buffer (Bruker, Rheinstetten, Germany). The samples were mixed gently, and 600 µL of the mixed sample were transferred into a 5 mm SampleJet rack tube (Bruker, Rheinstetten, Germany). Proton spectra were obtained at a constant temperature of 310 K using a standard nuclear Overhauser effect spectroscopy (NOESY) pulse sequence (Bruker: noesygppr1d), a Carr–Purcedll–Meiboom–Gill (CPMG) pulse sequence with presaturation during the relaxation delay (Bruker: cpmgpr1d) to achieve water suppression, and a standard 2D J-resolved (JRES) pulse sequence (Bruker: jresgpprqf) [9]. Data analysis was carried out using the Bruker IVDr Lipoprotein Subclass Analysis (B.I.LISA^TM^) method.

### 2.5. Metabolic Quantification Using NMR

To remove proteins and to quench enzymatic reactions in the samples, 200 µL serum was mixed with 400 µL methanol and stored at −20 °C for 1 h until further processing. Afterwards the samples were spun at 17,949 rcf at 4 °C for 30 min. Supernatants were lyophilized and 500 µL of NMR buffer in D_2_O were added to the samples and transferred to 5 mm NMR tubes. All NMR experiments were performed at 310 K on an AVANCE™ Neo Bruker Ultrashield 600 MHz spectrometer equipped with a TXI probe head and processed as described previously [10]. The 1D CPMG (Carr-Purcell_Meiboom_Gill) pulse sequence (cpmgpr1d, 512 scans, 73,728 points in F1, 11,904.76 HZ spectral width, 512 transients, recycle delays 4 s) with water suppression using pre-saturation, was used for ^1^H 1D NMR experiments. Bruker Topspin version 4.0.2 was used for NMR data acquisition. The spectra for all samples were automatically processed (exponential line broadening of 0.3 Hz), phased, and referenced using TSP at 0.0 ppm using Bruker Topspin 4.0.2 software (Bruker GmbH, Rheinstetten, Germany). Spectra pre-processing and data analysis was carried out using the state-of-the-art data analysis pipeline proposed by the group of Prof. Jeremy Nicholson at Imperials College London using Matlab^®^ scripts and MetaboAnalyst 4.0 [11]. NMR data were imported to Matlab^®^ vR2014a (Mathworks, Natick, MA, USA), regions around the water, TSP, and remaining methanol signals excluded, and probabilistic quotient normalization [12] was performed to correct for sample metabolite dilution; and reported concentrations corresponded to normalized concentrations.

### 2.6. Statistical Analyses

To identify changes in lipoprotein and metabolic profiles, multivariate statistical analysis was performed as described previously [13], and included principal component analysis (PCA), partial least squares—discriminant analysis (PLS-DA), orthogonal-partial least squares—discriminant analysis (O-PLS-DA) [14], with associated data consistency checks and 7-fold cross-validation, expressed by Q^2^. For the PCA and O-PLS-DA, NMR data were analyzed in Matlab^®^ vR2014a, and figures were prepared using MetaboAnalyst [11]. All other statistical analyses were performed with GraphPad Prism 8.0 (GraphPad Software, San Diego, CA, USA), SPSS 26 (SPSS Inc., Chicago, IL, USA), and Stata 15.0 (Stata Corp., Houston, TX, USA). Continuous variables were summarized as medians (25th–75th percentile), and categorical variables as absolute frequencies (%). Associations between variables were computed with cross-tabulations, Mann–Whitney U-tests, χ^2^-tests, and Fisher’s exact tests, as appropriate. Spearman’s rank-based correlation coefficient was used for correlation analyses. Formal adjustment for multiple testing was performed with the Sidak correction method. The prognostic associations between 28-day as well as ICU mortality and other potential baseline predictors were computed with univariable and multivariable logistic regression. Longitudinal trajectories of biomarker variables were analyzed with linear mixed models.

## 3. Results

### 3.1. Baseline Characteristics and Laboratory Results of the Study Population

In our study, 53 patients in the sepsis and 25 patients in the control cohort were included (Table 1). The median age of sepsis patients was 66 years (50–75), whereas ICU-survivors were 63 (46–76) and non-survivors 66 (53–73) years of age (*p* = 0.86), as previously reported [5]. In brief, females accounted for 40% of the sepsis study population and most infections (91%) were community-acquired. The primary focus of infection was the lung with 42%, followed by the abdomen with 17% and the urinary tract with 11%. Three, eight, and ten patients had received propofol, insulin, and glucose before sample acquisition, respectively. Parenteral nutrition was present in five patients at the time of sample acquisition, while zero patients had enteral nutrition. Samples were obtained in a median 3.3 h after ICU admission.

The baseline characteristics between sepsis ICU-survivors and non-survivors were mostly similar, but patients with septic shock compared to those without shock had higher PCT levels, higher serum creatinine levels, and higher SOFA scores, indicating higher disease severity. The ICU- and 28-day mortality of the sepsis cohort were 36 and 47%, respectively. In the ICU control cohort, e.g., patients with acute cardiovascular disease, cardiac arrest, intoxications, acute kidney injury, and other conditions, but without sepsis or bacteremia, the median age was 72 (65–79) years (*p* = 0.01 compared to sepsis cohort) with 60% being female. Despite being slightly older, ICU controls had similar rates of pre-existing diabetes or liver disease. Inflammatory markers including white blood count (WBC), CRP, and PCT were lower in ICU controls compared to sepsis patients.

### 3.2. Targeted Metabolomic Assessment of Lipoproteins in the Sepsis and Control Cohort

**Figure 1:**
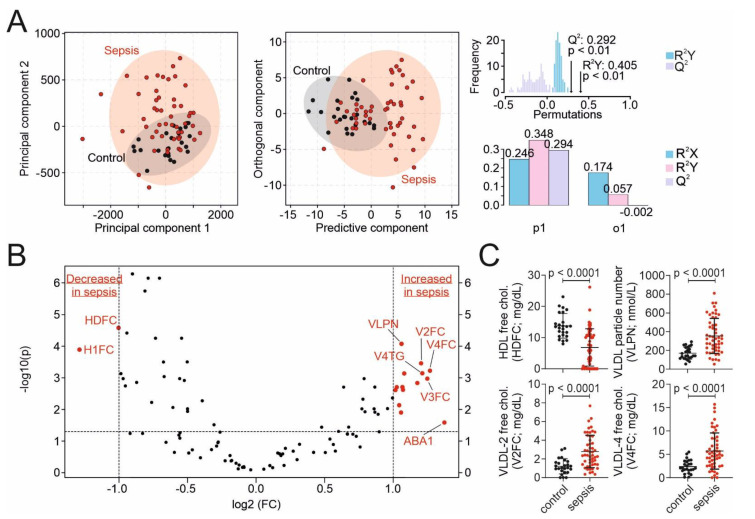
Targeted metabolomic assessment of lipoproteins. (**A**) Multivariate data analyses of lipoprotein parameters with principal component analyses (PCA) and orthogonal partial least squares discriminant analysis (O-PLS-DA) for the differentiation between ICU controls (black) and sepsis patients (red). PCA: principal component (PC) 1 of 76.8% and PC2 of 11.0%. O-PLS-DA: correlation coefficient R^2^Y = 0.405 and cross-validation score Q^2^ of 0.292 (*p* < 0.01). (**B**) Differences in lipoproteins between sepsis and control patients on a logarithmic scale (volcano plot). The vertical dashed lines mark the border to two-fold changes. The horizontal dashed line marks the level of significance at 0.05. (**C**) Boxplots of the most significant and most changed lipoproteins of sepsis (red color) and control (black color) patients. Abbreviations: HDFC = high density free cholesterol; H1FC = HDL-1 free cholesterol; VLDL = very-low-density lipoprotein; VLPN = VLDL particle number, V2FC = VLDL-2 free cholesterol; V3FC = VLDL-3 free cholesterol; V4FC = VLDL-4 free cholesterol; V4TG = VLDL-4 triglycerides; ABA1 = apolipoprotein B100 to apolipoprotein A-I ratio.

In a first step, targeted metabolomic assessment of lipoproteins using ^1^H NMR spectroscopy was performed to confirm and extend our previous results [5]. One patient in the sepsis cohort had to be excluded before lipoprotein analyses were performed due to insufficient sample volume in the aliquot. In the multivariate data analyses using PCA comparing the metabolomic lipoprotein profiles, we found differences between the sepsis and control group with a principal component 1 and 2 (PC1 and PC2) of 76.8% and 11.0%, respectively (Figure 1A). O-PLS-DA showed a separate clustering for sepsis and ICU control patients with a strong-moderate goodness of fit (correlation coefficient R^2^Y = 0.405) and a cross-validation score Q^2^ of 0.292 (*p* < 0.01, Figure 1A). Several lipoproteins were significantly different between the sepsis and the ICU control cohort (Table 2, Supplementary Table S1). In detail, patients in the sepsis compared to the ICU control group had, among other variables, significantly lower levels of HDL free cholesterol (HDFC), HDL cholesterol, and higher levels of very-low-density lipoprotein (VLDL) parameters (Figure 1B,C). In univariable logistic regression analyses for 28-day and ICU mortality, levels of most lipoproteins were not statistically significantly associated with outcomes (Supplementary Tables S2 and S3).

### 3.3. Untargeted Metabolomic Assessment of Metabolites in the Sepsis Cohort

**Figure 2:**
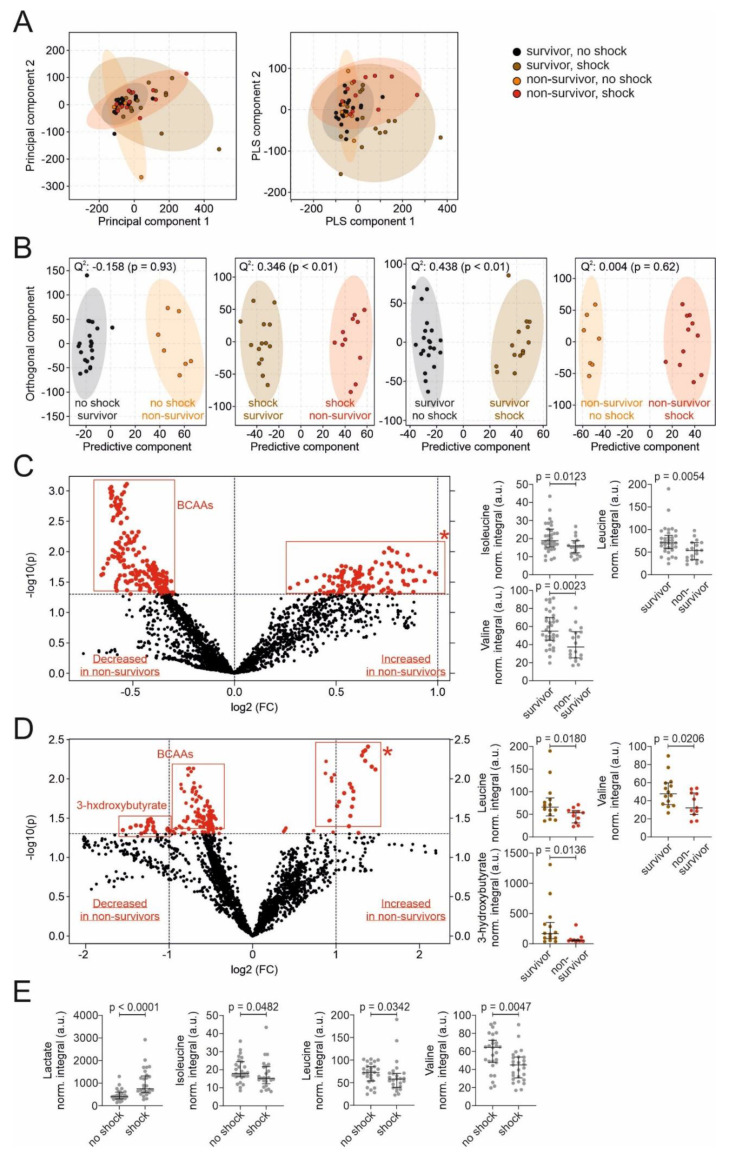
Untargeted metabolomic assessment of metabolites in sepsis. (**A**) Principal component analysis (PCA; left panel) and partial least squares discriminant analysis (PLS-DA; right panel) for the differentiation between the four groups of survivors and non-survivors, each for the shock and no-shock group. PCA: principal component (PC) 1 of 55% and PC2 of 14.3%. PLS-DA: Component 1 of 50.4%, Component 2 of 14.6%. (**B**) Pair-wise orthogonal partial least squares discriminant analysis (O-PLS-DA) showing acceptable clustering for survivors and non-survivor in the shock group (middle-left panel; Q^2^ = 0.346, *p* < 0.01), as well as survivors of the no-shock and shock group (middle-right panel; Q^2^ = 0.438, *p* < 0.01). No significant clustering was found for survivors and non-survivor in the no-shock group (left panel; *p* = 0.93) and for non-survivors of the no-shock and shock group (right panel; *p* = 0.62). (**C**) Differences in metabolites of all sepsis patients between survivors and non-survivors (volcano plot). The horizontal dashed line marks the level of significance at 0.05. The boxplots represent the most pronounced and most significant different metabolites. (**D**) Differences in metabolites of septic shock patients between survivors and non-survivors (volcano plot). The horizontal dashed line marks the level of significance at 0.05. The boxplots represent the most pronounced and most significant different metabolites. (**E**) Comparison between no-shock and septic shock patients. Boxplots of the most pronounced and most significant different metabolites. Abbreviations: BCAA = branched-chain amino acids (consisting of valine, leucine, and isoleucine). a.u. = arbitrary unit. * = unassigned metabolites.

In a second step, we extracted metabolites from serum samples to further investigate differences between sepsis and septic shock patients, as well as septic survivors and non-survivors. This approach allows for an untargeted metabolomic analysis to assess for differences of metabolic phenotypes between groups, which is more challenging in a complex blood sample matrix containing high concentrations of lipoproteins, masking metabolite signals. Moreover, a higher number of metabolites can be identified in this approach. In PCA analysis, we found differences between the four groups of survivors and non-survivors each for the shock and no-shock group with a PC1 of 55% and PC2 of 14.3% (Figure 2A). Pair-wise O-PLS-DA comparisons showed acceptable clustering for survivors and non-survivors in the shock group and for the comparison of no-shock and shock in the survivor group with cross-validation scores Q^2^ of 0.346 and 0.438, respectively (each *p* < 0.01, Figure 2B).

Blood serum levels of the branched-chain amino acids (BCAA) valine (33.0 vs. 55.0; *p* = 0.002), leucine (53.4 vs. 70.8; *p* = 0.005) and isoleucine (15.2 vs. 18.1; *p* = 0.012) were significantly lower in ICU non-survivors compared to ICU survivors (Table 3, Figure 2C). In the shock group, BCAA and 3-hydroxybutyrate (3-HB) were lower in non-survivors compared to survivors, while levels of other unassignable metabolites were higher in non-survivors (Figure 2D). Similarly, valine (43.3 vs. 64.3; *p* = 0.005), leucine (57.0 vs. 73.0; *p* = 0.034), and isoleucine (15.2 vs. 17.9; *p* = 0.048) levels were lower in shock patients compared to no-shock patients. Lactate, a marker in the definition of septic shock, served as an internal control, and levels were, as expected, higher in septic shock patients compared to those without shock (Figure 2E). In a sub-analysis, after the exclusion of patients who received propofol or renal replacement therapy (RRT), the results for the corresponding metabolite analyses remained similar.

### 3.4. Correlations between Metabolites and Other Parameters in the Sepsis Cohort

Higher SOFA scores as a marker for more severe organ dysfunction significantly inversely correlated with valine (r = −0.338, *p* = 0.01), isoleucine (r = −0.284, *p* = 0.04), but not leucine (−0.220, *p* = 0.114; Table 4). Valine correlated with other BCAA and inversely correlated with PCT (r = −0.401, *p* = 0.003) and CRP (r = −0.271, *p* = 0.05). Both leucine and isoleucine inversely correlated with PCT, but not CRP.

### 3.5. Univariable and Multivariable Regression Analyses

**Figure 3:**
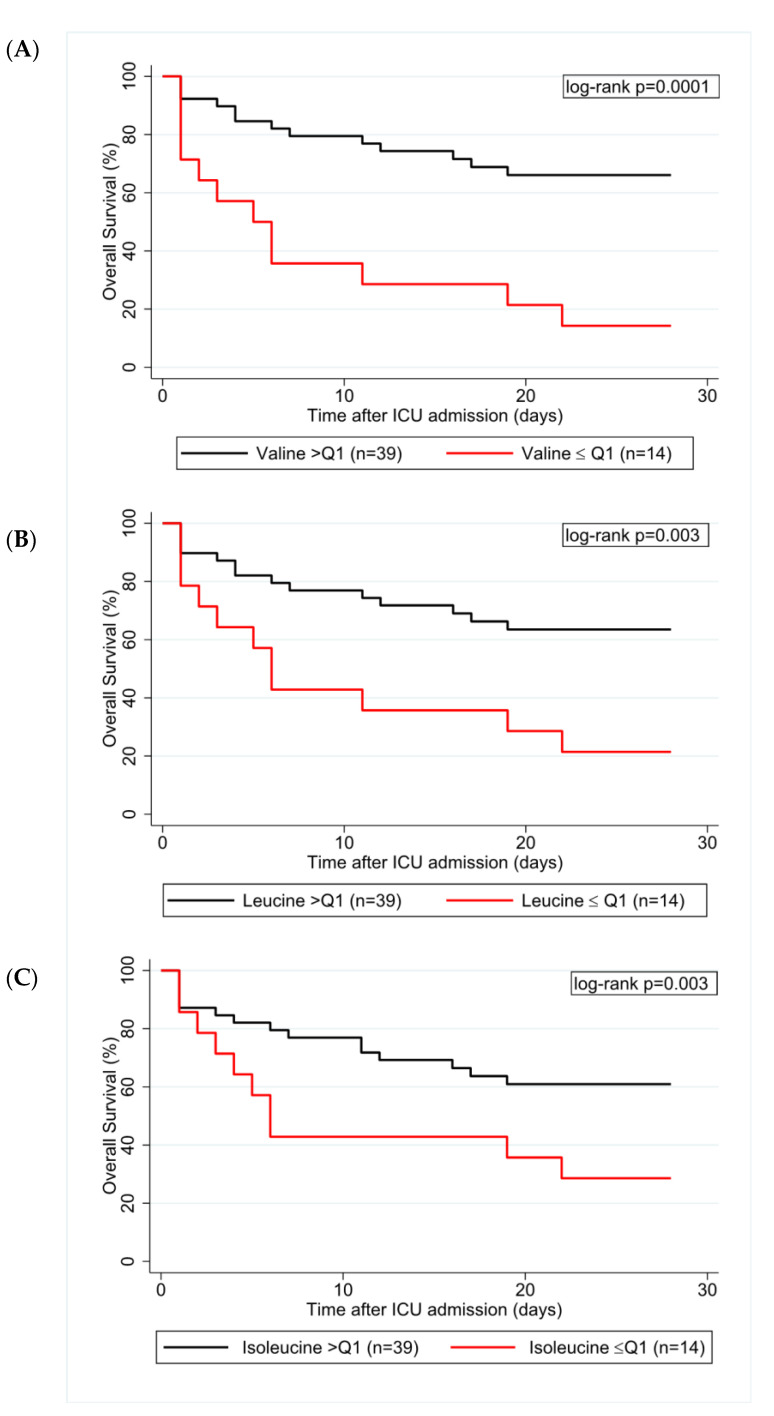
Kaplan–Meier estimates for valine (**A**), leucine (**B**), and isoleucine (**C**). The Q1, i.e., the 25th percentile, corresponds to a cutoff value for valine, leucine, and isoleucine of 33.3, 44.1, and 13.7 NSI units, respectively. The 28-day Kaplan–Meier survival estimates for patients below or above the respective cutoff were 14% vs. 66% for valine, 21% vs. 64% for leucine, and 29% vs. 61% for isoleucine. Abbreviations: Q1 = 25th percentile of the respective branched-chain amino acid, ICU = intensive care unit.

Univariable logistic regression was performed both for 28-day and ICU-mortality. In the sepsis cohort, for ICU-mortality only SOFA score and BCAA serum levels were significantly predictive for the outcome (Table 5). A lower SOFA score as wells as higher valine levels (odds ratio (OR) per doubling = 0.19, 95% CI: 0.06–0.58, *p* = 0.004), higher leucine levels (OR per doubling = 0.22, 95% CI: 0.07–0.66, *p* = 0.007), and higher isoleucine levels (OR per doubling = 0.23, 95% CI: 0.07–0.81, *p* = 0.023) were associated with reduced ICU-mortality. Similarly, several variables partly predicted 28-day mortality in our sepsis cohort. Older age, higher CRP, lower valine, leucine, and isoleucine serum levels were associated with increased 28-day mortality. In multivariable analyses, valine levels remained predictive for ICU and 28-day mortality (Table 6; Supplementary Tables S4 and S5). The 28-day Kaplan–Meier survival estimates for patients below the 25th percentile compared to those above this cutoff were 14% vs. 66% for valine (log-rank *p* = 0.0001), 21% vs. 64% for leucine (log rank *p* = 0.003) and 29% vs. 61% for isoleucine (log-rank *p* = 0.003; Figure 3).

### 3.6. Area under the Receiver Operating Characteristics (AUROC) Curve

**Figure 4:**
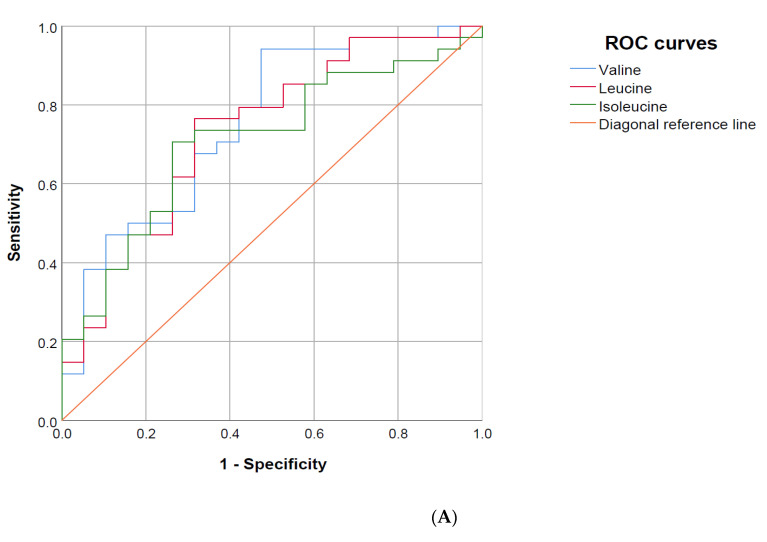

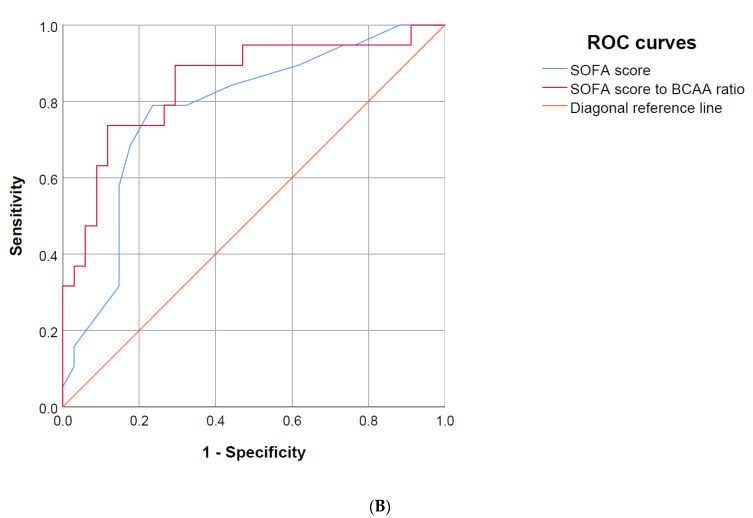
Area under the receiver operating characteristics (AUROC) of branched-chain amino acids for ICU-mortality. (**A**): AUROC for valine (blue line, 0.75 [0.62–0.89]), leucine (red line, 0.73 (0.59–0.88)) and isoleucine (green line, 0.71 (0.57–0.85)). (**B**): AUROC for the sequential organ failure assessment (SOFA) score (blue line, 0.78 (0.65–0.91)) and the SOFA-score to branched-chain amino acid ratio (red line, 0.85 (0.73–0.96)). The orange line is the diagonal reference line (50% chance).

The SOFA score and all three BCAA were strong discriminators regarding ICU-mortality (Table 7, Figure 4A). The BCAAs were furthermore strong discriminators for 28-day mortality, while the SOFA score was a weak discriminator for 28-day mortality in our cohort. In an exploratory analysis, a SOFA score to BCAA ratio was an even stronger discriminator of ICU-mortality (Figure 4B).

### 3.7. Longitudinal Data during the ICU Stay for Sepsis Patients

**Figure 5:**
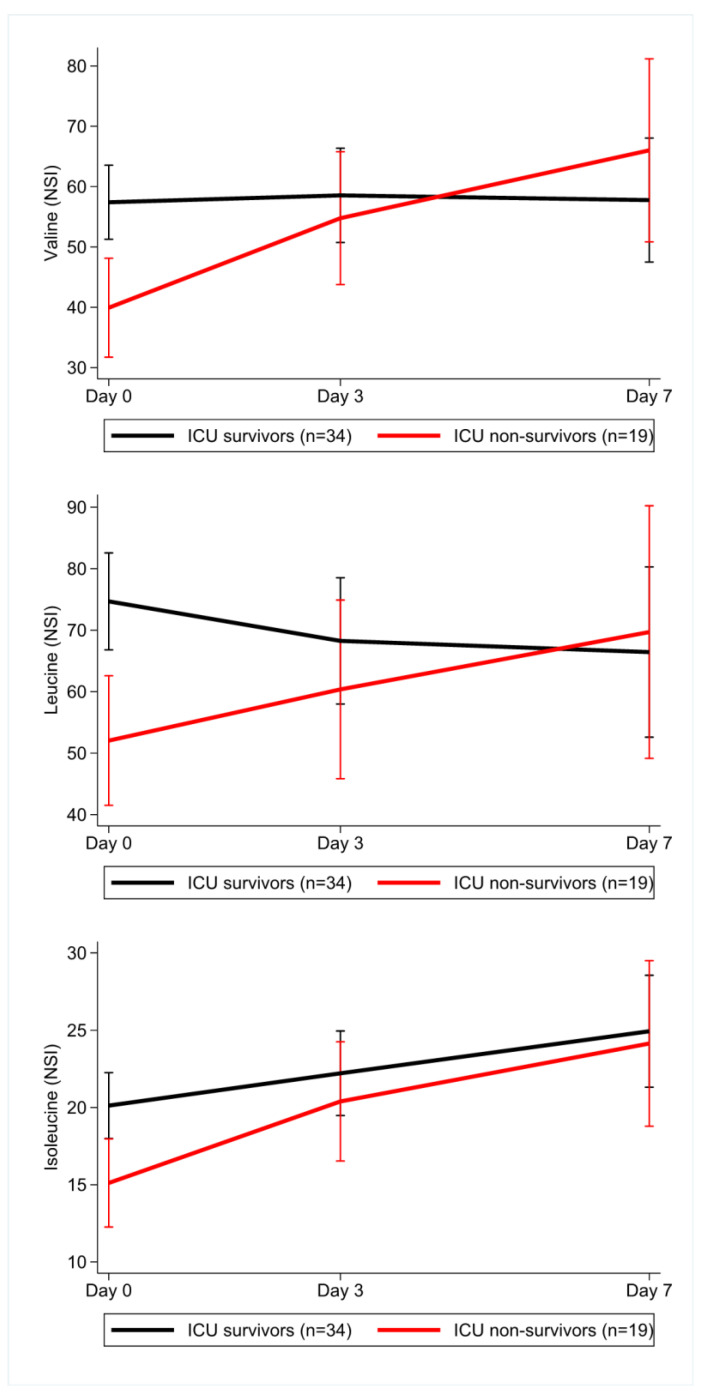
Longitudinal data of branched-chain amino acid levels. Mean levels of branched-chain amino acids (top panel: valine; middle panel: leucine; bottom panel: isoleucine) over time in sepsis survivors (black line) and sepsis non-survivors (red line) from day 0 to day 3 and 7 after admission. Survivors compared to non-survivors had significantly higher levels of branched-chain amino acids on day 0. Note that in the non-survivor cohort, lower branched-chain amino acids were associated with decreased time until death.

Samples on day three and day seven after ICU admission were available for 30 and 16 patients, respectively. To assess for potential bias, we compared patients in whom samples from day three or seven were available to those without samples on these days (already discharged or deceased) but found that the groups were not statistically significantly different (Supplementary Table S6). In a linear mixed model for the BCAAs we found that ICU survivors compared to non-survivors had similar values in our cohort over time. The ICU non-survivor subgroup got enriched over time with patients having higher valine levels, suggesting that higher valine levels may be associated with prolonged time to death in patients succumbing on ICU (Figure 5).

## 4. Discussion

In our study, we assessed the metabolomic profiles of patients with sepsis and septic shock who were admitted to the ICU and ICU patients without sepsis or bacteremia. Sepsis and septic shock are common in intensive care medicine and have high mortality rates [15,16]. The diagnosis is based on clinical criteria and includes a suspected infection by the treating physician and the presence of new organ dysfunctions [3]. The SOFA score is used to semi-quantitatively assess the severity of organ dysfunction and serves as a prognostic outcome marker. Several biomarkers in sepsis have been investigated without convincing results regarding the prognostic and diagnostic potential [17]. Established therapies in sepsis include early source control and early anti-infective therapy, while specific anti-inflammatory or pro-inflammatory therapies have failed [18]. Better prognostic biomarkers and therapeutic targets are urgently needed. In this study, we used ^1^H NMR spectroscopy to perform both a targeted analysis for lipoproteins as well as an untargeted approach to investigate a broad spectrum of metabolites simultaneously. Therefore, metabolites that were previously unknown to play a role may be detected. Furthermore, metabolomics, in contrast to genomics or proteomics, reflects the metabolites present at a given timepoint that are responsible for direct or indirect effects in the human body. Metabolites represent key molecules reflecting dietary and disease related pathway dysfunctions and may therefore provide an effective prognostic value and possible target for therapeutic intervention.

We were able to show in our study that valine, an essential amino acid, which must be consumed by protein-rich dietary products, was significantly reduced in non-survivors as compared to sepsis survivors. In addition, non-survivors compared to ICU survivors had lower levels of leucine and isoleucine. These three metabolites comprise the branched-chain amino acids (BCAA) group and are needed for protein synthesis and muscle strength. BCAA moreover inhibit proteolysis, improve immune function, and activate mTOR pathways [19,20,21]. Previously, higher levels of valine were found in diabetic humans with insulin resistance [22,23]. Insulin resistance leads to decreased glucose utilization and inhibited lipolysis, resulting in muscle protein degradation [24]. In our study, levels of valine were lower with increasing sepsis severity, despite insulin resistance and protein degradation, with skeletal muscle wasting being a common feature of septic patients [25,26]. Therefore, the BCAA catabolism must exceed the increased levels caused by insulin resistance and protein breakdown [19]. Another possible explanation for the low valine levels in sepsis may be an increased branched-chain alpha-keto acid dehydrogenase (BCKD) activity leading to increased oxidation of BCAA [27]. Liver failure or dysfunction is unlikely to play a role, as BCAA metabolism occurs primarily in extrahepatic tissues [28,29]. In our study, BCAA levels were furthermore not correlated with liver parameters such as bilirubin. As we cannot provide pre-admission laboratory values, it may be possible that people with low BCAA levels have a higher risk of developing sepsis and, in addition, have a worse prognosis when ICU admission is necessary. However, animal studies suggest that BCAA levels significantly decrease during LPS stimulation [30,31] and other small studies in humans also found decreased levels in sepsis patients [32,33]. Therefore, a decrease during sepsis instead of pre-existing low BCAA levels is more likely to account for the majority of BCAA scarcity, especially as the SOFA score as a marker for organ dysfunction is inversely correlated with valine and isoleucine.

In general, our data are mostly consistent with other studies showing reduced BCAA levels in sepsis, including a small study by Freund et al. that demonstrated higher levels of BCAA in sepsis survivors compared to non-survivors [24]. In addition, Puskarich et al. have found BCAA to be predictors for shock resolution in sepsis [34]. In another study comparing septic shock patients with and without encephalopathy, those with encephalopathy had higher levels of ammonia, phenylalanine, tryptophan, GABA, urea, and lower levels of isoleucine, cysteine, glutamine, and arginine [35]. In a more recent study by Liu et al., levels of ketone bodies, phenylalanine, glutamate, lactate, and others were significantly different between septic shock survivors and non-survivors, but no results on BCAA were provided [36]. Mickiewicz et al. investigated septic shock patients and found elevated levels of phenylalanine, lactate, 3-HB, as well as reduced levels of BCAAs and other metabolites compared to ICU controls [37,38]. In another study, valine, beta-hydroxybutyrate, citrate, and other metabolites in trauma patients were found to be predictors for later sepsis development [39]. In our study, we also showed reduced levels of BCAA in patients suffering from sepsis with lower levels in patients suffering from more severe disease. Furthermore, in the shock group, BCAA and 3-HB were lower in non-survivors compared to survivors. We did, however, only investigate sepsis patients for those metabolites, which may explain the differences in our study compared to the study by Mickiewicz and colleagues. On the other hand, lactate was one of the significantly different metabolites between shock and no-shock sepsis patients, which is based on the current diagnostic criteria for septic shock, and therefore provided an internal validation of our data. Su et al. found lower values of lactitol dehydrate and S-phenyl-D-cysteine and increased values of S-(3-methylbutanoyl)-dihydrolipoamide-E and N-nonanoyl-glycine in sepsis compared to SIRS patients [40]. S-(3-methylbutanoyl)-dihydrolipoamide is relevant for the degradation of BCAAs, which may explain the increased levels in sepsis patients [6]. The same group found in another study that the BCAA to aromatic amino acids (BCAA/AAA) ratio was significantly lower in sepsis patients compared to ICU controls [33]. The BCAA/AAA ratio is calculated in dividing (valine + leucine + isoleucine) by (phenylalanine + tyrosine) and is also called Fischer’s ratio [41]. BCAA may enhance ammonia detoxification in muscles, and BCAA supplementation improves hepatic encephalopathy in liver cirrhosis [42,43]. The current guidelines for nutrition in the ICU do not mention BCAA, while nutritional guidelines recommend BCAA in liver cirrhosis patients [44,45]. In sepsis patients, those with septic encephalopathy were found to have a decreased BCAA/AAA ratio [46]. A study investigating different concentrations of BCAA in parenteral nutrition solutions found a higher BCAA content to be correlated with a more positive nitrogen balance and an increased BCAA/AAA ratio [29]. Data regarding mortality endpoints are not consistent, but studies in animals and humans found beneficial effects of BCAA rich nutrition [47,48,49,50]. We also assessed the BCAA/AAA ratio and found comparable results, but the prognostic value did not further increase compared to solely investigating BCAA. However, the AUROC curves in our study showed promising results and further improvement of the predictive value when dividing the SOFA score by BCAA. In addition, when assessing longitudinal data of sepsis patients, we found that non-survivors with lower BCAA levels died earlier than those having higher, but compared to survivors decreased, levels of BCAA.

The reason for a poor prognosis in sepsis patients with low BCAA levels is probably multifaceted. It may be partially driven by a pre-existing reduced muscle mass which leads to fewer reserves in critical illness, but as discussed before, it likely only accounts for a minor aspect. In addition, we cannot rule out alterations of enzymes such as the branched-chain alpha-keto acid dehydrogenase complex (BCKDC), which is a multienzyme located at the inner mitochondrial membrane. BCAA may be metabolized through an increased BCKDC activity during several forms of stress especially being present in septic shock [27,51]. Low BCAA levels may also constitute a surrogate parameter for gastrointestinal dysfunction leading to low uptake of essential amino acids in critically ill patients. Indeed, we found a correlation between SOFA score and BCAA, suggesting that the levels are decreased with increased organ dysfunction.

BCAA, with its anticatabolic effects, may provide a therapeutic target as shown in a study by Shirabe et al., where bacteremia rates were reduced in liver transplant recipients receiving oral BCAA supplements [52]. Studies have also shown beneficial effects of BCAA supplementation in several sarcopenic cohorts, including the elderly and patients with chronic liver disease comprising those with cirrhosis [53,54,55,56,57,58]. Furthermore, BCAA enhances muscle strength and mass [54,59,60,61,62]. A well-known risk factor for worse sepsis outcomes is sarcopenia [63,64,65]. Therefore, the supplementation of BCAA in sepsis patients may especially be essential in those with sarcopenia. Whether a BCAA supplementation containing all three amino acids or whether only a leucine supplementation may be beneficial in sepsis needs to be investigated in future studies [66]. In contrast to non-critically ill patients, the uptake from the intestine may be reduced in ICU patients, so a parenteral route leads to a higher bioavailability compared to oral supplementation.

The limitation of many previous metabolomic sepsis studies is the use of a targeted (i.e., known metabolites) approach, while in our study, an untargeted ^1^H NMR technique was used for the investigation of metabolites. However, to allow for early prognosis prediction and to provide an early target for intervention, results of BCAA levels must be immediately available from central hospital laboratories, or close cooperation with metabolomic institutes with fast turn-around times is necessary. In our investigation, samples were extracted without lipoproteins to allow for the detection of small differences between groups, which may otherwise be lost to background noise. In general, data quality in the assessment of metabolites improves when lipoproteins are removed from samples. However, we additionally performed a targeted lipoprotein NMR approach and found altered levels of lipoproteins with lower HDL parameters in patients with sepsis compared to ICU controls. Still, lower quantitative lipoprotein levels were not associated with mortality outcomes. These results, analyzed with an independent method, confirm the findings of our previous study showing quantitative changes of lipoproteins to be less relevant than qualitative alterations [5].

Our study has several limitations: First, serum samples were used, and whole blood may provide even broader metabolomic results [67]. Second, we only included patients who were admitted to the ICU and earlier sampling points may be useful to predict the outcome. However, the presentation of patients within different times of the disease is a ubiquitous problem of sepsis studies. Furthermore, it must be noted that even an untargeted broad metabolomics approach is only a snapshot at a given time point. Third, another limitation of our study is sample size which limits external validity, and only the largest differences between groups may have been detected. Therefore, results need to be confirmed in larger studies. Fourth, we were not able to assess the type or amount of any recent oral food intake, as patients were critically ill, with many having an altered mental state on admission. Nevertheless, non-survivors had lower BCAA levels which may be used as a severity marker and may be investigated in future studies as a therapeutic target, independently of the history of food intake.

In conclusion, metabolomics using ^1^H NMR spectroscopy is a promising field as part of a personalized medical approach providing robust and standardizable results, especially as sample preparation effort is low. Lipoproteins are significantly different in sepsis patients compared to ICU patients without sepsis or bacteremia. Furthermore, we were able to identify BCAA (valine, leucine, and isoleucine) as significantly different metabolites in sepsis survivors when compared to non-survivors, and these amino acids may be biomarkers for early outcome prediction. In the future, BCAA may be investigated as a target for therapeutic interventions.

## Figures and Tables

**Table 1:** Demographics, disease severity, and patient outcomes in the sepsis and control cohort.

Variables	Sepsis Patients (N = 53)	Controls (N = 25)	*p*-Value
**Demographics & Premedication**			
Age (years)	66 (50–75)	72 (65–79)	0.012
Female sex	21 (40%)	15 (60%)	0.144
Anti-diabetic medication	12 (23%)	8 (32%)	0.413
Pre-existing diabetes	15 (28%)	8 (32%)	0.793
Pre-existing liver disease	3 (6%)	2 (8%)	0.653
**Disease severity and patient outcomes**			
SOFA score (points)	9 (7–13)	5 (3–9)	<0.0001
Presence of shock	26 (49%) #	7 (28%) *	0.079
28-day mortality	25 (47%)	4 (16%)	0.011
ICU mortality	19 (36%)	4 (16%)	0.110

SOFA = sequential organ failure assessment, ICU = intensive care unit. * Necessity of vasopressors and lactate >2 mmol/l. # according to sepsis-3 definition. Please also refer to Reisinger et al. Front Med.2020 [5].

**Table 2:** Lipid parameters in ICU sepsis patients and ICU controls assessed by targeted metabolomic analyses.

Variables	Sepsis Patients (N = 52)	Controls (N = 25)	*p*-Value	Below Sidak-Threshold *
**Main classes**				
Triglycerides (mg/dL)	185 (129–310)	101 (87–157)	<0.001	yes
Total cholesterol (mg/dL)	117 (106–148)	143 (119–194)	0.011	no
LDL cholesterol (mg/dL)	57 (39–76)	77 (53–106)	0.012	no
HDL cholesterol (mg/dL)	20 (13–30)	41 (32–51)	<0.001	yes
Total ApoA1 (mg/dL)	72 (52–98)	120 (98–139)	<0.001	yes
Total ApoA2 (mg/dL)	19 (15–23)	24 (20–27)	<0.001	no
Total ApoB100 (mg/dL)	82 (63–103)	74 (59–87)	0.171	no
LDL to HDL ratio	2.6 (1.7–3.7)	1.9 (1.5–2.5)	0.009	no
ApoB100 to ApoA1 ratio	1.3 (0.7–1.7)	0.6 (0.5–0.8)	<0.001	yes
**Particles**				
Total particle number (nmol/L)	1494 (1149–1877)	1338 (1063–1588)	0.171	no
VLDL particle number (nmol/L)	324 (205–490)	142 (118–233)	<0.001	yes
IDL particle number (nmol/L)	157 (79–300)	87 (61–137)	0.002	no
LDL particle number (nmol/L)	930 (737–1225)	1028 (720–1254)	0.640	no
**Triglycerides in subclasses**				
VLDL (mg/dL)	94 (63–199)	50 (43–111)	0.010	no
IDL (mg/dL)	13 (7–31)	6 (3–15)	0.007	no
LDL (mg/dL)	32 (19–58)	22 (17–30)	0.006	no
HDL (mg/dL)	15 (10–19)	13 (10–16)	0.124	no
**Cholesterol in subclasses**				
VLDL (mg/dL)	28 (20–41)	17 (12–24)]	<0.001	no
IDL (mg/dL)	21 (11–37)	11 (7–16)	0.002	no
LDL (mg/dL)	57 (39–76)	77 (53–106)	0.012	no
HDL (mg/dL)	20 (13–30)	41 (32–51)	<0.001	yes
**Free cholesterol in subclasses**				
VLDL (mg/dL)	13 (10–20)	8 (6–13)	0.002	no
IDL (mg/dL)	6 (3–11)	3 (2–4)	<0.001	no
LDL (mg/dL)	24 (19–34)	29 (21–39)	0.107	no
HDL (mg/dL)	6 (1–11)	14 (11–17)	<0.001	yes
**Phospholipids in subclasses**				
VLDL (mg/dL)	22 (15–41)	15 (11–28)	0.039	no
IDL (mg/dL)	5 (3–11)	4 (2–6)	0.133	no
LDL (mg/dL)	40 (28–55)	51 (34–62)	0.095	no
HDL (mg/dL)	36 (18–51)	62 (49–73)	<0.001	yes
**Apolipoproteins in subclasses**				
ApoA1 in HDL (mg/dL)	67 (46–98)	119 (98–138)	<0.001	yes
ApoA2 in HDL (mg/dL)	20 (17–25)	25 (21–27)	0.006	no
ApoB in VLDL (mg/dL)	18 (11–27)	8 (7–13)	<0.001	yes
ApoB in IDL (mg/dL)	9 (4–17)	5 (3–8)	0.002	no
ApoB in LDL (mg/dL)	51 (41–67)	57 (40–69)	0.640	no

* *p*-Value corrections for multiple testing were performed with the Sidak-method (lower values than the threshold are significant)—Sidak-Treshold at 0.00044984. Data are reported as medians (25th–75th percentile). Abbreviations: ICU = intensive care unit; VLDL = very low-density lipoprotein; LDL = low-density lipoprotein; IDL = intermediate-density lipoprotein; HDL = high-density lipoprotein; ApoA1 = apolipoprotein A-I; ApoA2 = apolipoprotein A-II; ApoB100 = apolipoprotein B-100.

**Table 3:** Assigned and most prominent metabolic changes with sepsis and septic shock determined by untargeted ^1^H NMR spectroscopy.

Variable	Sepsis Cohort N = 53	Shock N = 26	No-Shock N = 27	*p*-Value	ICU-Survivors N = 34	Non-Survivors N = 19	*p*-Value
**Metabolomic results**							
Valine	49.7 (33.3–65.6)	43.3 (29.0–53.7)	64.3 (47.7–72.3)	0.005	55.0 (44.8–70.2)	33.0 (24.9–53.9)	0.002
Leucine	65.3 (44.1–81.3)	57.0 (38.4–71.0)	73.0 (54.3–86.3)	0.034	70.8 (57.8–87.5)	53.4 (32.0–71.0)	0.005
Isoleucine	17.0 (13.7–22.4)	15.2 (10.9–21.6)	17.9 (16.1–24.4)	0.048	18.1 (14.6–24.6)	15.2 (11.1–17.9)	0.012
Acetate *	35.6 (27.2–44.3)	35.8 (30.4–48.4)]	30.9 (24.5–44.1)	0.292	34.8 (29.5–46.4)	35.6 (24.1–48.6)	1.000
3-Hydroxybutyrate	93.4 (49.8–166.6)	86.0 (47.5–208.9)	93.4 (58.3–159.7)	1.000	98.2 (57.8–205.3)	64.2 (47.9–133.9)	0.221
Phenylalanine	33.3 (23.7–47.4)	35.6 (25.8–48.0)	27.9 (21.9–47.3)	0.188	30.0 (23.6–47.4)	36.0 (24.2–48.2)	0.458
Tyrosine	8.1 (6.3–11.3)	8.2 (6.2–12.6)	8.1 (6.3–11.3)	0.715	8.7 (7.0–11.5)	7.0 (5.6–9.3)	0.156
Lactate	587 (383–914)	815 (586–1394)	409 (301–601)	< 0.0001	566 (392–995)	587 (347–890)	0.970
Citrate	22.8 (19.1–28.3)	24.3 (18.6–29.7)	22.5 (19.2–25.5)	0.466	22.7 (19.1–28.7)	23.5 (19.1–27.3)	0.970

* 52 values. Note that all results are displayed as normalized signal intensity (NSI) units.

**Table 4:** Heatmap of correlations of selected variables and metabolites in the sepsis cohort.

Variables	Age	Valine	Leucine	Isoleucine	Acetate	3-HB	Phenyl-alanine	Tyrosine	Citrate	Lactate	BMI	CRP	PCT	IL−6
SOFA	−0.188	−0.338 *	−0.220	−0.284 *	−0.226	−0.219	0.078	−0.021	0.004	0.198	0.136	0.097	0.378 **	0.344 *
Age		−0.050	−0.042	0.078	0.011	0.184	0.037	−0.097	0.001	−0.012	0.041	0.224	−0.141	−0.069
Valine			0.860 **	0.833 **	0.321 *	0.145	−0.319 *	0.566 **	−0.004	−0.009	0.084	−0.271 *	−0.401 **	−0.394 **
Leucine				0.798 **	0.310 *	0.298 *	−0.304 *	0.448 **	0.059	−0.002	−0.052	−0.223	−0.396 **	−0.375 **
Isoleucine					0.215	0.303 *	−0.271 *	0.564 **	0.108	0.021	0.043	−0.208	−0.408 **	−0.270
Acetate						0.194	−0.109	0.287 *	−0.124	0.320 *	−0.010	−0.428 **	−0.219	−0.292 *
3-HB							−0.053	−0.017	−0.082	0.045	0.039	0.006	−0.276 *	−0.130
Phenylalanine								0.004	0.206	0.041	0.218	0.035	0.267	0.263
Tyrosine									0.006	0.436 **	0.160	−0.476 **	−0.125	−0.024
Citrate										0.090	0.048	0.055	−0.112	0.096
Lactate											0.149	−0.114	0.120	0.268
BMI												−0.040	0.036	0.015
CRP													0.255	0.375 **
PCT														0.450 **

Abbreviations: 3-HB = 3-Hydroxybutyrate; BMI = body mass index; CRP = C-reactive protein; IL-6 = interleukin-6; PCT = procalcitonin; SOFA = sequential organ failure assessment. * = *p*-value < 0.05; ** = *p*-value < 0.01. Dark red: very strong correlation (+/− >0.65); Red: strong correlation (+/− 0.55–0.65); Orange: moderate correlation (+/− 0.45–0.55); Light blue: weak correlation (+/− 0.35–0.45); Blue: very weak correlation (+/− 0.25–0.35); Dark blue: no correlation (+/− <0.25).

**Table 5:** Univariable logistic regression models in sepsis patients for prediction of 28-day and ICU mortality.

Outcome Variable	28-Day Mortality			ICU Mortality		
Variable	Odds Ratio	95% Confidence Interval	*p*	Odds Ratio	95% Confidence Interval	*p*
**Demographics**						
Age (per 5 years increase)	1.23	1.02–1.50	0.033	1.06	0.89–1.27	0.511
Female sex	2.71	0.87–8.42	0.085	1.65	0.53–5.17	0.390
Anti-diabetic therapy	0.75	0.20–2.75	0.665	0.87	0.22–3.37	0.836
Type 2 diabetes	0.45	0.13–1.57	0.210	0.56	0.15–2.08	0.384
Liver disease	2.35	0.20–27.6	0.497	3.88	0.33–45.93	0.282
**Laboratory parameters**						
White blood count (per 1 G/L increase)	1.02	0.98–1.07	0.357	1.00	0.96–1.05	0.960
Hemoglobin (per 1 g/dL increase)	0.94	0.77–1.15	0.574	1.03	0.84–1.25	0.801
Platelets (per 100 G/L increase)	1.11	0.71–1.75	0.640	1.14	0.71–1.81	0.593
C-reactive protein (per 100 mg/L increase)	1.72	1.07–2.77	0.025	1.40	0.90–2.18	0.136
Procalcitonin (per 10 ng/mL increase)	1.03	0.97–1.09	0.339	1.04	0.98–1.11	0.170
Serum creatinine (per 1 mg/dL increase)	1.01	0.85–1.19	0.905	1.04	0.88–1.23	0.653
Serum bilirubin (per 1 mg/dL increase)	0.89	0.74–1.08	0.245	0.94	0.80–1.11	0.484
**Metabolites**						
Valine (per doubling)	0.18	0.06–0.56	0.003	0.19	0.06–0.58	0.004
Leucine (per doubling)	0.19	0.06–0.59	0.004	0.22	0.07–0.66	0.007
Isoleucine (per doubling)	0.29	0.09–0.93	0.038	0.23	0.07–0.81	0.023
Acetate (per doubling)	1.26	0.57–2.80	0.572	1.24	0.54–2.85	0.609
3-Hydroxybutyrate (per doubling)	0.91	0.61–1.38	0.668	0.79	0.50–1.26	0.326
Phenylalanine (per doubling)	1.77	0.75–4.19	0.194	1.23	0.53–2.88	0.631
Tyrosine (per doubling)	0.83	0.35–1.95	0.665	0.82	0.33–2.04	0.675
Lactate (per doubling)	1.15	0.65–2.04	0.632	1.00	0.55–1.81	0.996
Citrate (per doubling)	1.41	0.44–4.54	0.563	0.90	0.27–3.03	0.865
**Sepsis severity score**						
SOFA score (per 1 point increase)	1.13	0.97–1.31	0.113	1.36	1.12–1.65	0.002

Abbreviations: SOFA = sequential organ failure assessment. The odds ratio per doubling of the predictor variable was obtained by a log_2_(x + 1) transformation of the variable. Note that metabolites are measured in normalized signal intensity units.

**Table 6:** Multivariable logistic regression models in sepsis patients for the prediction of 28-day and ICU mortality.

**Multivariable Model 1: 28-Day Mortality**	**Odds Ratio**	**95% Confidence Interval**	***p***
Age (per 5 year increase)	1.25	1.00–1.56	0.048
C-reactive protein (per 100 mg/L increase)	1.37	0.80–2.35	0.257
Valine (per doubling)	0.19	0.05–0.66	0.009
**Multivariable Model 2: ICU Mortality**	**Odds Ratio**	**95% Confidence Interval**	***p***
SOFA score (per 1 point increase)	1.29	1.06–1.57	0.012
Valine (per doubling)	0.26	0.08–0.85	0.026

Abbreviations: ICU = intensive care unit. SOFA = sequential organ failure assessment. The odds ratio per doubling of the predictor variable were obtained by a log_2_(x + 1) transformation of the variable.

**Table 7:** ROC curves for 28-day and ICU mortality.

Outcome	ICU Mortality		28-day Mortality	
Variables	AUROC	95% Confidence Interval	AUROC	95% Confidence Interval
SOFA score	0.78	0.65–0.91	0.62	0.46–0.78
Valine	0.75	0.62–0.89	0.75	0.62–0.89
Leucine	0.73	0.59–0.88	0.75	0.62–0.88
Isoleucine	0.71	0.57–0.85	0.69	0.54–0.83
SOFA score/BCAA-ratio	0.85	0.73–0.96	0.74	0.60–0.88

Abbreviations: AUROC = area under the receiver operating curve. ICU = intensive care unit. SOFA = sequential organ failure assessment. BCAA = branched-chain amino acids (valine + leucine + isoleucine).

## Data Availability

The data presented in this study are available on request from the corresponding author.

## Supplementary Materials

The following are available online at https://www.mdpi.com/article/10.3390/nu13093106/s1, Table S1: Lipoprotein parameters in ICU sepsis patients and ICU controls, Table S2: Univariable logistic regression for lipoproteins (sepsis cohort), Table S3: Univariable logistic regression for lipoproteins (ICU controls), Table S4: Additional multivariable model for 28-day and ICU mortality including other BCAA, Table S5: Additional multivariable model for 28-day mortality including the SOFA score, Table S6: Baseline characteristics in longitudinal analyses.
