# Supplementary material for: Branched-Chain Amino Acids Can Predict Mortality in ICU Sepsis Patients
Source: Nutrients. 2021 Sep 3;13(9):3106. doi: 10.3390/nu13093106 (PMC8469152; doi:10.3390/nu13093106)
Supplement: Supplementary file 1 [file nutrients-13-03106-s001.zip › nutrients-1350829-supplementary.pdf]

Branched-chain amino acids predict mortality in ICU sepsis patients

Short title: Metabolomics in sepsis

Alexander C. Reisinger, MD; Florian Posch, PhD; Gerald Hackl, MD; Gunther Marsche, PhD; Harald Sourij, MD; Benjamin Bourgeois, PhD; Kathrin Eller, MD; Tobias Madl, PhD; Philipp Eller, MD

Supplementary tables

Supplementary Table S1: Lipoprotein parameters in ICU sepsis patients and ICU controls

Variables		Sepsis patients (N=52)	Controls (N=25)	p-Value	Below Sidak-treshold*
**VLDL subfraction - triglycerides**					
VLDL 1 triglycerides (mg/dL)		36.1 [14.4-88.1]	26.9 [14.3-54.8]	0.184	no
VLDL 2 triglycerides (mg/dL)		17.8 [7.3-28.7]	11.2 [6.9-16.8]	0.069	no
VLDL 3 triglycerides (mg/dL)		19.0 [10.5-28.9]	10.9 [6.9-16.1]	0.001	no
VLDL 4 triglycerides (mg/dL)		19.7 [13.0-29.3]	10.2 [6.5-14.3]	<0.001	yes
VLDL 5 triglycerides (mg/dL)		5.3 [3.9-6.6]	3.6 [2.8-4.5]	<0.001	yes
**VLDL subfraction - cholesterol**					
VLDL 1 cholesterol (mg/dL)		7.0 [3.7-11.5]	4.4 [2.9-8.3]	0.082	no
VLDL 2 cholesterol (mg/dL)		3.3 [1.4-5.3]	2.2 [1.1-3.3]	0.061	no
VLDL 3 cholesterol (mg/dL)		4.9 [3.0-8.2]	3.3 [1.5-4.2]	0.004	no
VLDL 4 cholesterol (mg/dL)		9.5 [4.0-14.2]	5.1 [2.7-6.2]	<0.001	no
VLDL 5 cholesterol (mg/dL)		2.0 [1.2-3.4]	1.3 [0.7-2.0]	0.003	no
**VLDL subfraction – free cholesterol**					
VLDL 1 free cholesterol (mg/dL)		1.0 [0.0-4.0]	0.9 [0.0-2.6]	0.300	no
VLDL 2 free cholesterol (mg/dL)		2.4 [1.4-4.3]	1.1 [0.6-1.7]	<0.001	yes
VLDL 3 free cholesterol (mg/dL)		3.1 [1.7-4.4]	1.4 [0.8-2.3]	<0.001	yes
VLDL 4 free cholesterol (mg/dL)		4.9 [2.7-8.2]	2.1 [1.5-3.5]	<0.001	yes
VLDL 5 free cholesterol (mg/dL)		1.0 [0.6-1.8]	0.7 [0.2-1.1]	0.007	no
**VLDL subfraction – phospholipids**					
VLDL 1 phospholipids (mg/dL)		4.7 [1.4-10.2]	3.9 [1.6-7.5]	0.601	no
VLDL 2 phospholipids (mg/dL)		3.6 [1.0-6.1]	2.9 [1.3-3.6]	0.149	no
VLDL 3 phospholipids (mg/dL)		5.3 [3.2-8.6]	3.0 [1.5-4.6]	0.003	no
VLDL 4 phospholipids (mg/dL)		9.1 [5.8-13.9]	4.7 [3.4-6.6]	<0.001	yes
VLDL 5 phospholipids (mg/dL)		2.5 [1.8-3.6]	2.0 [1.2-2.5]	0.023	no
**Continued on next page**					

continued		Sepsis patients (N=52)	Controls (N=25)	p-Value	Below Sidak-treshold*
**LDL subfractions**					
LDL 1 particle number (nmol/L)		260 [118-439]	209 [137-317]	0.453	no
LDL 2 particle number (nmol/L)		193 [151-248]	170 [116-255]	0.507	no
LDL 3 particle number (nmol/L)		182 [148-254]	207 [99-253]	0.939	no
LDL 4 particle number (nmol/L)		104 [36-156]	119 [53-233]	0.281	no
LDL 5 particle number (nmol/L)		46 [0-124]	119 [60-162]	0.005	no
LDL 6 particle number (nmol/L)		146 [11-240]	186 [116-253]	0.187	no
**LDL subfraction - triglycerides**					
LDL 1 triglycerides (mg/dL)		13.4 [8.7-23.6]	7.8 [5.8-10.8]	<0.001	yes
LDL 2 triglycerides (mg/dL)		4.0 [2.6-7.4]	2.7 [1.7-4.0]	0.005	no
LDL 3 triglycerides (mg/dL)		3.5 [2.9-5.6]	3.1 [2.2-4.1]	0.030	no
LDL 4 triglycerides (mg/dL)		3.4 [1.7-7.5]	3.1 [1.4-3.7]	0.093	no
LDL 5 triglycerides (mg/dL)		2.2 [0.7-4.2]	1.7 [1.1-2.5]	0.824	no
LDL 6 triglycerides (mg/dL)		4.0 [3.0-6.0]	3.7 [2.8-4.4]	0.279	no
**LDL subfraction - cholesterol**					
LDL 1 cholesterol (mg/dL)		19.1 [6.3-31.4]	16.7 [10.2-26.9]	0.983	no
LDL 2 cholesterol (mg/dL)		14.2 [10.1-18.1]	15.6 [9.7-20.6]	0.453	no
LDL 3 cholesterol (mg/dL)		14.2 [9.7-18.0]	17.1 [7.3-21.9]	0.415	no
LDL 4 cholesterol (mg/dL)		4.9 [0.0-9.9]	9.2 [3.0-18.6]	0.020	no
LDL 5 cholesterol (mg/dL)		0.4 [0.0-4.6]	7.7 [2.6-11.6]	0.001	no
LDL 6 cholesterol (mg/dL)		6.6 [0.0-11.7]	10.6 [6.1-14.8]	0.019	no
**LDL subfraction – free cholesterol**					
LDL 1 free cholesterol (mg/dL)		6.2 [2.9-10.2]	6.0 [3.7-8.6]	0.909	no
LDL 2 free cholesterol (mg/dL)		5.0 [3.8-6.6]	5.7 [3.4-7.5]	0.405	no
LDL 3 free cholesterol (mg/dL)		4.8 [2.8-6.8]	5.1 [3.1-7.5]	0.483	no
LDL 4 free cholesterol (mg/dL)		2.5 [0.7-3.9]	3.3 [2.0-6.4]	0.034	no
LDL 5 free cholesterol (mg/dL)		1.3 [0.0-2.7]	3.2 [1.4-4.4]	<0.001	no
LDL 6 free cholesterol (mg/dL)		2.0 [0.0-3.6]	3.6 [2.8-4.9]	<0.001	no
**LDL subfraction – phospholipids**					
LDL 1 phospholipids (mg/dL)		13.0 [5.4-20.8]	11.1 [7.1-16.3]	0.761	no
LDL 2 phospholipids (mg/dL)		8.3 [6.6-10.8]	9.2 [5.8-12.3]	0.624	no
LDL 3 phospholipids (mg/dL)		8.0 [5.8-11.0]	10.3 [4.6-13.0]	0.466	no
LDL 4 phospholipids (mg/dL)		3.3 [0.7-6.2]	5.5 [2.4-10.5]	0.042	no
LDL 5 phospholipids (mg/dL)		0.9 [0.0-3.9]	4.9 [2.8-6.8]	<0.001	no
LDL 6 phospholipids (mg/dL)		4.2 [0.1-6.8]	6.7 [4.7-9.6]	0.014	no
**LDL subfraction – ApoB**					
LDL 1 phospholipids (mg/dL)		14.3 [6.5-24.2]	11.5 [7.5-17.5]	0.453	no
LDL 2 phospholipids (mg/dL)		10.6 [8.3-13.7]	9.4 [6.4-14.0]	0.507	no
LDL 3 phospholipids (mg/dL)		10.0 [8.1-14.0]	11.4 [5.5-13.9]	0.939	no
LDL 4 phospholipids (mg/dL)		5.7 [2.0-8.6]	6.5 [2.9-12.8]	0.281	no
LDL 5 phospholipids (mg/dL)		2.5 [0.0-6.8]	6.5 [3.3-8.9]	0.006	no
LDL 6 phospholipids (mg/dL)		8.0 [0.6-13.2]	10.2 [6.4-13.9]	0.187	no
**Continued on next page**					

continued		Sepsis patients (N=52)	Controls (N=25)	p-Value	Below Sidak-treshold*
**HDL subfraction – triglycerides**					
HDL 1 triglycerides (mg/dL)		5.4 [3.5-7.7]	4.1 [2.6-5.3]	0.104	no
HDL 2 triglycerides (mg/dL)		2.5 [1.8-3.2]	2.1 [1.5-2.9]	0.167	no
HDL 3 triglycerides (mg/dL)		2.6 [1.7-3.2]	2.2 [1.7-2.7]	0.236	no
HDL 4 triglycerides (mg/dL)		4.0 [3.1-4.8]	3.6 [2.7-4.3]	0.161	no
**HDL subfraction – cholesterol**					
HDL 1 cholesterol (mg/dL)		6.6 [0.0-12.3]	11.3 [8.2-17.8]	<0.001	yes
HDL 2 cholesterol (mg/dL)		3.2 [0.9-4.9]	5.8 [4.4-6.8]	<0.001	yes
HDL 3 cholesterol (mg/dL)		4.3 [2.2-7.0]	7.4 [5.9-8.1]	<0.001	yes
HDL 4 cholesterol (mg/dL)		9.2 [5.8-13.7]	14.3 [9.8-20.2]	0.003	no
**HDL subfraction – free cholesterol**					
HDL 1 free cholesterol (mg/dL)		0.4 [0.0-3.2]	4.1 [2.5-5.4]	<0.001	yes
HDL 2 free cholesterol (mg/dL)		1.2 [0.6-1.7]	1.8 [1.4-2.4]	<0.001	yes
HDL 3 free cholesterol (mg/dL)		0.7 [0.2-1.6]	1.8 [1.5-2.3]	<0.001	yes
HDL 4 free cholesterol (mg/dL)		2.8 [1.4-4.4]	3.5 [2.7-5.0]	0.026	no
**HDL subfraction – phospholipids**					
HDL 1 phospholipids (mg/dL)		8.8 [0.1-17.6]	16.0 [10.7-23.0]	<0.001	no
HDL 2 phospholipids (mg/dL)		6.6 [3.3-8.9]	9.9 [7.5-13.3]	<0.001	yes
HDL 3 phospholipids (mg/dL)		7.8 [5.3-11.2]	12.6 [9.3-14.1]	<0.001	yes
HDL 4 phospholipids (mg/dL)		14.1 [10.3-18.1]	20.4 [16.7-26.2]	<0.001	yes
**HDL subfraction – ApoA1**					
HDL 1 ApoA1 (mg/dL)		8.7 [0.0-20.6]	21.9 [12.1-27.2]	<0.001	yes
HDL 2 ApoA1 (mg/dL)		9.2 [4.9-12.9]	14.6 [11.8-18.7]	<0.001	yes
HDL 3 ApoA1 (mg/dL)		14.7 [10.5-21.0]	22.2 [18.3-24.3]	<0.001	yes
HDL 4 ApoA1 (mg/dL)		40.8 [29.1-51.8]	56.1 [47.6-79.2]	<0.001	yes
**HDL subfraction – ApoA2**					
HDL 1 ApoA2 (mg/dL)		1.4 [0.1-2.1]	2.0 [0.9-3.1]	0.032	no
HDL 2 ApoA2 (mg/dL)		2.1 [1.6-2.8]	2.2 [1.5-3.2]	0.539	no
HDL 3 ApoA2 (mg/dL)		4.0 [2.7-5.3]	4.4 [3.9-5.2]	0.221	no
HDL 4 ApoA2 (mg/dL)		11.4 [6.2-15.3]	14.3 [11.4-18.6]	0.014	no

* p-Value corrections for multiple testing were performed with the Sidak-method (lower values than the threshold are significant) - Sidak-Treshold at 0.00044984.

Supplementary Table S2: Univariable logistic regression for lipoproteins (sepsis cohort):

Outcome variable	28-day mortality				ICU mortality		
**Variable**	**Odds ratio**	**95% confidence intervall**	**p**		**Odds ratio**	**95% confidence intervall**	**p**
**Main classes**							
Triglycerides (mg/dL)	0.78	0.37-1.62	0.501		0.90	0.42-1.93	0.789
Total cholesterol (mg/dL)	0.44	0.15-1.25	0.121		0.43	0.15-1.21	0.108
LDL cholesterol (mg/dL)	0.90	0.59-1.37	0.629		0.85	0.56-1.30	0.456
HDL cholesterol (mg/dL)	0.92	0.53-1.58	0.756		0.85	0.49-1.49	0.576
Total ApoA1 (mg/dL)	0.92	0.46-1.85	0.824		0.74	0.36-1.52	0.417
Total ApoA2 (mg/dL)	0.23	0.07-0.78	0.018		0.37	0.14-0.99	0.047
Total ApoB100 (mg/dL)	0.60	0.20-1.78	0.357		0.67	0.22-2.06	0.485
**Particles**							
Total particle number (nmol/L)	0.60	0.21-1.76	0.356		0.67	0.22-2.03	0.482
VLDL particle number (nmol/L)	0.91	0.48-1.72	0.765		1.02	0.52-2.00	0.947
IDL particle number (nmol/L)	0.71	0.45-1.12	0.144		0.77	0.51-1.17	0.223
LDL particle number (nmol/L)	0.75	0.35-1.63	0.473		0.78	0.35-1.74	0.550
**Triglycerides in subclasses**							
VLDL (mg/dL)	0.94	0.57-1.58	0.826		0.95	0.56-1.63	0.857
IDL (mg/dL)	0.87	0.59-1.28	0.475		0.94	0.62-1.40	0.749
LDL (mg/dL)	0.73	0.38-1.40	0.339		0.88	0.45-1.71	0.697
HDL (mg/dL)	1.03	0.49-2.17	0.928		1.09	0.49-2.41	0.838
**Cholesterol in subclasses**							
VLDL (mg/dL)	0.69	0.36-1.32	0.259		0.72	0.37-1.42	0.347
IDL (mg/dL)	0.73	0.45-1.16	0.184		0.78	0.49-1.23	0.826
LDL (mg/dL)	0.90	0.60-1.37	0.629		0.85	0.56-1.30	0.456
HDL (mg/dL)	0.92	0.53-1.58	0.756		0.85	0.49-1.49	0.576
**Free cholesterol in subclasses**							
VLDL (mg/dL)	0.80	0.42-1.52	0.499		0.79	0.41-1.54	0.495
IDL (mg/dL)	0.73	0.43-1.25	0.257		0.81	0.47-1.40	0.457
LDL (mg/dL)	1.01	0.55-1.85	0.985		1.02	0.54-1.94	0.955
HDL (mg/dL)	1.03	0.72-1.45	0.861		0.90	0.62-1.31	0.594
**Phospholipids in subclasses**							
VLDL (mg/dL)	1.05	0.71-1.55	0.811		1.01	0.67-1.52	0.962
IDL (mg/dL)	0.77	0.49-1.20	0.244		0.87	0.55-1.37	0.551
LDL (mg/dL)	0.87	0.40-1.90	0.731		0.89	0.40-1.99	0.773
HDL (mg/dL)	1.07	0.66-1.74	0.777		1.02	0.62-1.69	0.942
**Apolipoproteins in subclasses**							
ApoA1 in HDL (mg/dL)	1.09	0.78-1.54	0.613		1.00	0.71-1.42	0.991
ApoA2 in HDL (mg/dL)	0.20	0.05-0.74	0.016		0.34	0.12-1.00	0.049
ApoB in VLDL (mg/dL)	0.89	0.44-1.77	0.732		1.01	0.49-2.08	0.979
ApoB in IDL (mg/dL)	0.66	0.38-1.15	0.147		0.75	0.43-1.30	0.310
ApoB in LDL (mg/dL)	0.75	0.34-1.65	0.475		0.78	0.34-1.77	0.556

Regression results per doubling were obtained by using a log2 transformed variable.

Abbreviations: ICU=intensive care unit; VLDL = very low-density lipoprotein; LDL = low-density lipoprotein; IDL = intermediate density lipoprotein; HDL = high-density lipoprotein; ApoA1 = Apolipoprotein A-I; ApoA2 = Apolipoprotein A-II; ApoB100 = Apolipoprotein B-100

Supplementary Table S3: Univariable logistic regression for lipoproteins (ICU controls):

Outcome variable	28-day mortality				ICU mortality		
**Variable**	**Odds ratio**	**95% confidence intervall**	**p**		**Odds ratio**	**95% confidence intervall**	**p**
**Main classes**							
Triglycerides (mg/dL)	0.31	0.04-2.45	0.270		0.31	0.04-2.45	0.270
Total cholesterol (mg/dL)	1.47	0.15-14.06	0.740		1.47	0.15-14.06	0.740
LDL cholesterol (mg/dL)	1.58	0.30-8.38	0.594		1.58	0.30-8.38	0.594
HDL cholesterol (mg/dL)	1.13	0.09-13.82	0.922		1.13	0.09-13.82	0.922
Total ApoA1 (mg/dL)	3.34	0.10-112.81	0.502		3.34	0.10-112.81	0.502
Total ApoA2 (mg/dL)	2.45	0.13-47.08	0.553		2.45	0.13-47.08	0.553
Total ApoB100 (mg/dL)	1.03	0.08-13.32	0.984		1.03	0.08-13.32	0.984
**Particles**							
Total particle number (nmol/L)	1.03	0.08-12.95	0.982		1.03	0.08-12.95	0.982
VLDL particle number (nmol/L)	0.22	0.03-1.58	0.131		0.22	0.03-1.58	0.131
IDL particle number (nmol/L)	0.88	0.29-2.71	0.823		0.88	0.29-2.71	0.823
LDL particle number (nmol/L)	1.81	0.25-13.31	0.560		1.81	0.25-13.31	0.560
**Triglycerides in subclasses**							
VLDL (mg/dL)	0.37	0.08-1.82	0.222		0.37	0.08-1.82	0.222
IDL (mg/dL)	0.70	0.31-1.58	0.396		0.70	0.31-1.58	0.396
LDL (mg/dL)	1.77	0.22-14.21	0.592		1.77	0.22-14.21	0.592
HDL (mg/dL)	0.54	0.04-7.39	0.641		0.54	0.04-7.39	0.641
**Cholesterol in subclasses**							
VLDL (mg/dL)	0.49	0.12-2.08	0.335		0.49	0.12-2.08	0.335
IDL (mg/dL)	0.94	0.34-2.63	0.907		0.94	0.34-2.63	0.907
LDL (mg/dL)	1.58	0.30-8.38	0.594		1.58	0.30-8.38	0.594
HDL (mg/dL)	1.13	0.09-13.82	0.922		1.13	0.09-13.82	0.922
**Free cholesterol in subclasses**							
VLDL (mg/dL)	0.25	0.03-2.48	0.236		0.25	0.03-2.48	0.236
IDL (mg/dL)	0.79	0.19-3.22	0.744		0.79	0.19-3.22	0.744
LDL (mg/dL)	2.66	0.31-22.61	0.370		2.66	0.31-22.61	0.370
HDL (mg/dL)	3.06	0.16-58.83	0.458		3.06	0.16-58.83	0.458
**Phospholipids in subclasses**							
VLDL (mg/dL)	0.41	0.08-2.16	0.293		0.41	0.08-2.16	0.293
IDL (mg/dL)	0.79	0.29-2.15	0.639		0.79	0.29-2.15	0.639
LDL (mg/dL)	2.20	0.30-16.08	0.438		2.20	0.30-16.08	0.438
HDL (mg/dL)	3.50	0.16-74.77	0.422		3.50	0.16-74.77	0.422
**Apolipoproteins in subclasses**							
ApoA1 in HDL (mg/dL)	2.47	0.09-66.18	0.591		2.47	0.09-66.18	0.591
ApoA2 in HDL (mg/dL)	2.27	0.08-61.44	0.626		2.27	0.08-61.44	0.626
ApoB in VLDL (mg/dL)	0.18	0.02-1.74	0.138		0.18	0.02-1.74	0.138
ApoB in IDL (mg/dL)	0.80	0.17-3.68	0.771		0.80	0.17-3.68	0.771
ApoB in LDL (mg/dL)	1.82	0.24-13.81	0.563		1.82	0.24-13.81	0.563

Regression results per doubling were obtained by using a log2 transformed variable.

Note that ICU and 28-day mortality are identical in the ICU control cohort.

Abbreviations: ICU=intensive care unit; VLDL = very low-density lipoprotein; LDL = low-density lipoprotein; IDL = intermediate density lipoprotein; HDL = high-density lipoprotein; ApoA1 = Apolipoprotein A-I; ApoA2 = Apolipoprotein A-II; ApoB100 = Apolipoprotein B-100.

Supplementary Table S4: Additional multivariable model for 28-day and ICU mortality including other BCAA

**Multivariable model 1: 28-day mortality**		**Odds ratio**	**95% confidence intervall**	**p**
Age (per 5 year increase)		1.24	1.00-1.55	0.049
C-reactive protein (per 100 mg/dL increase)		1.39	0.80-2.40	0.240
Leucine (per doubling)		0.20	0.06-0.69	0.011
**Multivariable model 1: 28-day mortality**		**Odds ratio**	**95% confidence intervall**	**p**
Age (per 5 year increase)		1.27	1.02-1.58	0.034
C-reactive protein (per 100 mg/dL increase)		1.50	0.90-2.51	0.122
Isoleucine (per doubling)		0.26	0.07-0.98	0.047
**Multivariable model 2: ICU mortality**		**Odds ratio**	**95% confidence intervall**	**p**
SOFA score (per 1 point increase)		1.33	1.09-1.62	0.005
Leucine (per doubling)		0.27	0.09-0.83	0.022
**Multivariable model 2: ICU mortality**		**Odds ratio**	**95% confidence intervall**	**p**
SOFA score (per 1 point increase)		1.31	1.08-1.59	0.006
Isoleucine (per doubling)		0.35	0.09-1.27	0.111

Supplementary Table S5: Additional multivariable model for 28-day mortality including the SOFA score

Multivariable model 1: 28-day mortality		Odds ratio	95% confidence intervall	p
Age (per 5 year increase)		1.33	1.03-1.71	0.028
C-reactive protein (per 100 mg/dL increase)		1.36	0.78-2.36	0.272
Valine (per doubling)		0.21	0.06-0.78	0.019
SOFA score (per 1 point increase)		1.14	0.94-1.38	0.185

Multivariable model 1: 28-day mortality		Odds ratio	95% confidence intervall	p
Age (per 5 year increase)		1.34	1.04-1.73	0.024
C-reactive protein (per 100 mg/dL increase)		1.32	0.76-2.32	0.326
Leucine (per doubling)		0.21	0.06-0.75	0.016
SOFA score (per 1 point increase)		1.18	0.97-1.43	0.100

Multivariable model 1: 28-day mortality		Odds ratio	95% confidence intervall	p
Age (per 5 year increase)		1.36	1.05-1.75	0.019
C-reactive protein (per 100 mg/dL increase)		1.47	0.86-2.49	0.156
Isoleucine (per doubling)		0.29	0.08-1.15	0.078
SOFA score (per 1 point increase)		1.17	0.97-1.42	0.099

Supplementary Table S6: Baseline characteristics in longitudinal analyses

Variable	No samples on day 3/7 n=21	Samples on day 3 n=30*	p-Value compared to no samples	Samples on day 7 n=16	p-Value compared to no samples
**Demographics**					
Age (years)	64 [52-77]	66 [49-74]	0.969	64 [47-73]	0.774
Female sex	9 (43%)	11 (37%)	0.650	5 (31%)	0.515
Liver Disease	2 (10%)	1 (3%)	0.561	1 (6%)	1.000
Type 2 diabetes	7 (33%)	8 (27%)	0.608	3 (19%)	0.461
Anti-diabetic therapy	4 (19%)	8 (27%)	0.739	3 (19%)	1.000
**Laboratory covariables**					
WBC (G/L)	13.6 [9.1-26.5]	16.8 [8.7-27.7]	0.774	16.5 [7.8-25.8]	0.797
Hemoglobin (g/dL)	9.5 [8.3-11.7]	11.1 [9.0-13.3]	0.110	11.1 [10.7-13.2]	0.051
Platelets (G/L)	149 [88-274]	166 [75-263]	0.738	168 [98-294]	0.476
C-reactive protein (mg/L)	204 [152-361]	217 [81-294]	0.379	196 [94-292]	0.404
Procalcitonin (ng/mL)	8.3 [2.1-65.3]	8.9 [0.6-35.0]	0.585	2.1 [0.4-30.1]	0.130
Serum bilirubin (mg/dL)	1.02 [0.59-2.32]	0.77 [0.43-2.77]	0.379	0.75 [0.40-1.63]	0.338
Serum creatinine (mg/dL)	2.9 [1.8-3.8]	2.3 [1.5-4.8]	0.745	1.7 [0.9-3.6]	0.123
**Sepsis severity and outcomes**					
SOFA score (points)	8 [7-14]	11 [8-13]	0.520	9 [9-13]	0.774
SAPS3 score (points)	65 [51-75]	68 [57-76]	0.348	66 [57-74]	0.705
28-day mortality	10 (48%)	14 (47%)	0.947	7 (44%)	0.815
ICU mortality	8 (38%)	10 (33%)	0.727	5 (31%)	0.739

Comparison of patients, in whom samples on day 3 or day 7 were available to those without obtained samples.
Data are reported as medians [25th-75th percentile], or absolute frequencies (%).
Abbreviations: ICU=intensive care unit, SAPS3 = Simplified acute physiology score 3
* Note that 2 patient samples were not available on day 3 but on day 7 (patients therefore alive until >d7)
